# Antenatal care addressing gestational weight gain (GWG): a cross sectional study of pregnant women’s reported receipt and acceptability of recommended GWG care and associated characteristics

**DOI:** 10.1186/s12884-023-06158-4

**Published:** 2024-02-06

**Authors:** Jenna L Hollis, Kristine Deroover, Milly Licata, Belinda Tully, Eva Farragher, Christophe Lecathelinais, Nicole Bennett, Michelle Foster, Craig E Pennell, John Wiggers, Justine Daly, Melanie Kingsland

**Affiliations:** 1 Hunter New England Population Health, Longworth Avenue, Locked Bag 10, Wallsend, NSW 2287 Australia; 2 School of Medicine and Public Health, College of Health, Medicine and Wellbeing, University of Newcastle, Callaghan, NSW 2308 Australia; 3 Population Health Research Program, Hunter Medical Research Institute, New Lambton Heights, NSW 2305 Australia; 4 Gomeroi Nation, New England North West, NSW Australia; 5 Hunter New England Local Health District Nursing and Midwifery Services, Newcastle, NSW 2305 Australia

**Keywords:** Pregnancy, GWG, Antenatal care, Maternal, Implementation, Evidence based practice

## Abstract

**Background:**

The Australian Clinical Practice Guidelines for Pregnancy Care recommend that during the first and subsequent antenatal visits all pregnant women are weighed; advised of recommended gestational weight gain (GWG), dietary intake and physical activity; and offered referrals for additional support if needed. The extent to which these recommendations are implemented and women’s acceptability of recommended care is unknown. This study examines women’s reported receipt and acceptability of guideline care for GWG, and characteristics associated with receipt of such care and its acceptability.

**Methods:**

From September 2018 to February 2019 a telephone survey was undertaken with women who had recently had a baby and received antenatal care from five public maternity services within a health district in Australia. Women self-reported their demographic characteristics, and receipt and acceptability of recommended GWG care. Receipt and acceptability of such care, and their association with the characteristics of women and the maternity service they attended, were examined using descriptive statistics and multivariable logistic regression analyses.

**Results:**

Of 514 women, 13.1% (95%CI:10.3–16.5) reported that they received an assessment of weight at both their first and a subsequent antenatal visit, and less than one third (30.0%; 95%CI:26.0-33.9) received advice on their recommended GWG range, dietary intake and physical activity. Just 6.6% (95%CI:4.8–9.1) of women reported receiving all assessment and advice components of recommended antenatal care, and 9.9% (95%CI:7.6–12.8) of women reported being referred for extra support. Women who were younger (OR = 1.13;95%CI:1.05–1.21), identifying as Aboriginal and Torres Strait Islander (OR = 24.54;95%CI:4.98-120.94), had a higher pre-pregnancy BMI (OR = 1.13;95%CI:1.05–1.21), were experiencing their first pregnancy (OR = 3.36;95%CI:1.27–8.86), and lived in a least disadvantaged area (compared to mid-disadvantaged area (OR = 18.5;95%CI:2.6-130.5) and most disadvantaged area (OR = 13.1;95%CI:2.09–82.4)) were more likely to receive recommended assessment and advice. Most Aboriginal (92%) and non-Aboriginal (93%) women agreed that recommended GWG care is acceptable.

**Conclusion:**

Most women perceive antenatal care for GWG as recommended by the Clinical Practice Guidelines as acceptable, but did not receive it. When provided, such care is not delivered consistently to all women regardless of their characteristics or those of the maternity service they attend. There is a need for service-wide practice change to increase routine GWG care in pregnancy for all women.

**Supplementary Information:**

The online version contains supplementary material available at 10.1186/s12884-023-06158-4.

## Introduction

Gestational weight gain (GWG) outside of recommended ranges is associated with an increased risk of pregnancy complications, poor perinatal outcomes, and obesity and chronic disease risk for the mother and child [1]. Despite such risks, up to 70% of pregnant women gain weight outside their recommended ranges [1]. Services that provide antenatal care are recognised as a key setting to support women to gain weight within recommended ranges, and engage in recommended dietary intake and physical activity behaviours during pregnancy [2, 3]. Routine weighing and monitoring of weight gain by antenatal care providers against the Institute of Medicine (IOM) weight gain ranges [4] is recommended in many countries including the United States, Canada and Australia [4, 5]. In Australia, the Clinical Practice Guidelines for Pregnancy Care recommend three elements of care for addressing GWG; assess, advise, and refer [6]. It is recommended that during the initial and subsequent antenatal visits all pregnant women have a weight assessment, and are advised of their recommended weight gain range, dietary intake and physical activity for pregnancy [6]. Women gaining weight at a rate outside of their recommended weight gain range are recommended to be offered referrals for additional support from specialist health professionals, such as a dietitian [6].

A narrative review of 54 studies assessing GWG communication between women and health care providers showed that GWG care was infrequent and often inaccurate [7]. Frequency of GWG advice ranged from 9.5% [8] to as high as 83% [9], and the accuracy of recommended GWG ranges reported by pregnant women ranged from 0 to 85% [7]. In another systematic review of 17 studies including 20,717 women, only 50% of women reported GWG advice consistent with the IOM guidelines [10]. Research has also shown that pregnant women with a low or high pre-pregnancy body mass index (BMI) are more likely to receive routine weight assessments [11–15], and women of older age, higher socioeconomic status, having their first child, with a history of dieting, low levels of physical activity and with a high BMI are more likely to receive GWG advice [7]. Few studies internationally [7, 10], and none in Australia, have comprehensively investigated the extent to which pregnant women receive *all* care elements (i.e. assess, advice and refer) of guideline recommended care for GWG as part of routine antenatal care. Also, few international studies and no Australian studies have comprehensively explored characteristics of pregnant women and antenatal services associated with receipt of recommended GWG care. Given these evidence gaps, there is a need to determine if recommended care is being delivered to all women regardless of their economic, social, and psychosocial determinants, or those of the maternity service that they attend.

Pregnant women’s low acceptability of GWG care has been reported by antenatal care providers as a barrier to the routine delivery of recommended care [7, 16, 17]. Antenatal care providers report a belief that pregnant women do not want to be weighed [16] and concern that it may cause unnecessary anxiety, resulting in a reluctance for antenatal care providers to weigh pregnant women [7, 16, 17]. In contrast, most women report wanting to receive care for GWG from their health care providers [11, 12, 18–21]. For example, in a study of 582 pregnant Australian women, 80% wanted education on weight gain, nutrition and physical activity, particularly at the beginning of pregnancy, with no difference in responses between women with a healthy weight or overweight pre-pregnancy BMI [21]. No studies have reported on the acceptability of all recommended GWG care elements by all pregnant women. An understanding of the acceptability of each GWG care element, and any personal or maternity service characteristics associated with low acceptability, is needed to maximise engagement by pregnant women.

The primary aim of this study was to examine pregnant women’s receipt and acceptability of guideline recommended antenatal care addressing GWG, and their associations with characteristics of pregnant women and of their maternity services. Secondary aims were to report (i) the prevalence of women with GWG within recommendations, (ii) their recall of their recommended GWG range, and (iii) women’s preference for mode of receiving GWG support.

## Materials and methods

### Ethical approval

The study was approved by the Hunter New England Human Research Ethics Committee (16/11/16.407), Aboriginal Health and Medical Research Council (1236/16), and the University of Newcastle Human Research Ethics Committee (H-2017-0032). All methods were carried out in accordance with relevant guidelines and regulations.

### Design, participants and recruitment

A cross sectional survey was undertaken between September 2018 and February 2019 with women who had recently had a baby and received antenatal care from five public maternity services from three sectors within a health district in New South Wales, Australia. The services were in metropolitan, regional and rural locations and provided antenatal care to 70% of women (over 6000 women annually) giving birth in the district’s public hospitals.

The services provided care for pregnant women through a range of antenatal care models, including: hospital and community-based midwifery clinics, midwifery group practice continuity of care, specialist medical clinics, Aboriginal Maternal and Infant Health Services (AMIHS) and multidisciplinary care for women with complex pregnancies or identified vulnerabilities. Antenatal care providers include registered midwives, medical practitioners, and Aboriginal Health Workers, and can be supported by a range of specialist health professionals including dietitians.

#### Pregnant women

To be eligible to participate in this study women needed to be at least 18 years of age, had attended any of the five public maternity services within the study area for antenatal care, and given birth at least one month (and not more than five months) prior. Women were ineligible to participate if their antenatal care was primarily provided through a private obstetrician, if they had experienced a negative pregnancy outcome (e.g. stillbirth or miscarriage), or could not comply with study procedures (e.g. unable to complete a telephone call in English).

#### Recruitment procedure

Participants were initially recruited to participate in a study during pregnancy relating to antenatal care for alcohol consumption in pregnancy [22] and had consented to be contacted for further research. Recruitment to the initial study involved all women attending public maternity services within the study area receiving written information at their first antenatal visit informing them of the study and that they may be contacted and invited to complete a survey throughout their antenatal care based on their attendance at the service. A toll free telephone number was provided in the information for women to call to decline participation and sampling for the survey. Electronic medical record and appointment data were used to generate a weekly sample of eligible women across the study area. Of the eligible women, 105 women were randomly sampled each week via a computerised random number generator and mailed an information statement explaining the purpose of the survey and inviting them to participate. One week later, non-Aboriginal women were followed up via a telephone call and invited to participate in a computer assisted telephone interview (CATI). Based on advice regarding a culturally appropriate survey approach, women who identified as Aboriginal or Torres Strait Islander and/or women attending an AMIHS for antenatal care received a text message after the information statement was mailed, and were provided with the option of completing the survey via CATI or online. At the end of the survey, women were asked for their consent to be contacted for further surveys relating to alcohol consumption, smoking, and healthy weight gain.

For this study, all women who had consented to be contacted for further research, who had a live birth and a baby aged between four and 21 weeks at the time of the survey were invited to participate. Potentially eligible women were mailed an information statement outlining the purpose of the study and inviting them to participate. A toll free telephone number was provided for women to decline study participation. Women who did not decline via the toll free telephone number, received up to 10 phone contact attempts within a two week period inviting them to participate. Verbal consent to participate was recorded by the CATI interviewer prior to beginning the survey. Eligibility criteria relating to their English language proficiency (being sufficient to complete the survey unaided) was assessed at commencement of the CATI. Women could decline participation at any stage during the telephone and online survey.

#### Data collection procedures

The survey questions were developed based on previous surveys conducted in antenatal care settings to assess women’s self-report receipt of care [12, 14, 23, 24] and according to recommended care elements for GWG outlined in the Clinical Practice Guidelines for Pregnancy Care [6]. The surveys were reviewed for cultural appropriateness for Aboriginal women and pilot tested prior to use. Study data were collected and managed using **REDCap** electronic data capture tools hosted at Hunter New England Population Health, NSW Health. Trained and experienced female interviewers conducted the CATI surveys with pregnant women. Aboriginal women were offered the option of an Aboriginal interviewer if preferred.

Data on the maternity service characteristics were obtained from electronic medical record and appointment systems. Participant demographic characteristics were collected during the initial alcohol care survey. Both maternity service and women’s demographic characteristic data were linked to individual participant data from the CATI survey. Additional pregnancy characteristics (including week’s gestation at the time of the first antenatal visit and birth, diagnosis of gestational diabetes mellitus, and singleton or multiple pregnancy) and an additional maternity service characteristics (antenatal care provider profession at first antenatal visit) were collected via the survey in the present study.

### Measures

#### Anthropometric measures

All women were asked to report their height (in centimetres or inches), and their weight (in kilograms or pounds/ounces) at a series of time points in pregnancy: pre-pregnancy, at their first antenatal visit, and immediately prior to birth.

#### Recall of recommended weight gain

Women were asked to report how much weight their antenatal care provider recommended that they gain during their pregnancy.

#### Receipt of antenatal care for GWG

Women answered survey items assessing whether they received care for GWG from their antenatal care provider during their first and subsequent antenatal visits according to recommended care elements (assess, advise and refer) [6].

- *Assess (first antenatal visit)*: women were asked if their height and weight were measured (and if not, whether an antenatal care provider offered to weigh them, but they declined), and if they were asked to report their pre-pregnancy weight (and if not, whether their pre-pregnancy weight had been reported in their medical record or from referral documentation).
- *Assess (subsequent antenatal visit/s)*: women were asked if they were weighed (including whether an antenatal care provider offered to weigh the woman, but they declined).
- *Advise*: women were asked to report if recommended GWG, and dietary intake and physical activity to support recommended weight gain were discussed during their antenatal visit.
- *Refer*: women were asked if they had been referred to any other service for additional GWG support, such as a dietitian.

The specific wording of the questions and possible response options are shown in Additional File 1.

#### Characteristics associated with care receipt

Data were collected on characteristics of pregnant women and maternity services that were hypothesised to be associated with the provision of recommended antenatal care for GWG.

- *Pregnant women’s characteristics*: age, Aboriginal or Torres Strait Islander status, index of relative socioeconomic disadvantage (a measure that ranks areas in Australia according to relative socio-economic advantage and disadvantage using women’s residential postal codes [25]), pre-pregnancy BMI, education level, weeks pregnant at first antenatal visit, first or subsequent pregnancy, singleton or multiple (e.g. twins) pregnancy, diagnosis of gestational diabetes mellitus, and model of antenatal care (to indicate pregnancy risk level).
- *Maternity service characteristics*: provider/s seen during antenatal visit, and maternity service geographical location.

#### Acceptability of antenatal care for GWG

Women’s acceptability of receiving assessment and care for GWG during antenatal visits was assessed through questions informed by a previous survey with pregnant women attending maternity services [24]. Women were asked if (i) maintaining a healthy weight during pregnancy was important to them, and (ii) whether it would be acceptable to be weighed and given advice on recommended weight gain, dietary intake and physical activity as routine antenatal care. Reponses were reported on a 5-point Likert scale (strongly agree, agree, neither agree nor disagree, disagree, strongly disagree). Women were then asked a series of statements to determine if there were circumstances or approaches that would make routine weighing and GWG advice more or less acceptable to them, including: if antenatal care providers felt it was important for providing care for their health and that of their baby; if the antenatal care provider asked the woman if they wanted to discuss the topic first; if the antenatal care provider approached the topic in a sensitive, non-judgement way; if weighing and advice was provided in a private room; and if they knew their antenatal care provider would support them with healthy weight gain throughout their pregnancy. Finally, women were asked how (i.e. mode of delivery) they would prefer to receive information and advice to support a healthy weight gain during their pregnancy (response options outlined in Additional File 1).

### Statistical analyses

Data analysis was conducted using SAS software, Version 9.3 [26]. Condensed response options were created for whether women identified as Aboriginal and/or Torres Strait Islander (‘Aboriginal or Torres Strait Islander or both’ or ‘Neither Aboriginal or Torres Strait Islander’), highest education level completed (‘Completed high school or less’ or ‘Completed technical certificate or diploma’ or ‘Completed university or college degree or higher’), and antenatal care providers seen in the visit (‘doctor only’ or ‘midwife and doctor’ or ‘midwife only’ or ‘other provider involved’).

The type and model of antenatal care the woman was receiving was used to indicate pregnancy risk level, with hospital and community-based midwifery clinics, midwifery group practice continuity of care and multidisciplinary care for women with social vulnerabilities used to classify ‘low risk pregnancy’, and specialist medical clinics and multi-disciplinary care for women with complex medical needs models used to classify ‘high risk pregnancy’. Women’s residential postal codes were used to determine socio-economic disadvantage using the Index of Relative Socio-economic Disadvantage (IRSD) [25] with index quintiles collapsed into ‘most disadvantaged’ (quintiles one and two), ‘mid disadvantaged’ (quintile three) and ‘least disadvantaged’ (quintiles four and five). Maternity service postal code was used to calculate the maternity service’s geographical remoteness (‘major city’ or ‘regional or rural’) using the Access/Remoteness Index of Australia [27]. Women’s reported acceptability of each of the care elements was dichotomised into ‘acceptable’ (strongly agree and agree) and ‘not acceptable’ (strongly disagree, disagree and unsure).

Pre-pregnancy weight and height were used to calculate pre-pregnancy BMI. BMI was classified using the World Health Organization equation and categories of underweight, healthy weight, overweight and obesity [28]. Total GWG during pregnancy was calculated by subtracting self-report pre-pregnancy weight from self-report weight prior to birth. GWG within recommendations was calculated based on the IOM guideline ranges for GWG based on pre-pregnancy BMI [4], adjusting for week’s gestation.

The accuracy of the recommended weight gain range that women recall being provided by their antenatal care provider was examined for singleton pregnancies, and calculated based on data collected for the following question: “How much total weight did the health professional recommended you gain during pregnancy? (report in kilograms or pounds)”, and compared to the IOM guideline ranges for GWG based on each woman’s pre-pregnancy BMI.

GWG care delivery dichotomous outcome variables were created. Participants who responded ‘I do not remember’ or ‘I would prefer not to answer’ were coded with ‘no’ responses.

- ‘Assessment at first and subsequent antenatal visits’: reported receipt of assessment at first antenatal visit and assessment at a subsequent visit;
- ‘Advice at first antenatal visit’: reported receipt of advice about recommended GWG range, dietary intake and physical activity; and.
- ‘All assessment and advice for GWG’: reported receipt of assessment at the first and a subsequent antenatal visit, and all advice elements at the first antenatal visit.

Descriptive statistics were used to describe pregnant women and maternity service characteristics, and receipt and acceptability of recommended care for GWG. Multivariable logistic regression analyses were used to examine associations between all characteristics (pregnant woman’s and maternity service characteristics) and (i) the receipt of antenatal care for GWG (4 models) and (ii) acceptability of antenatal care for GWG (1 model).

## Results

### Participants

Over the six month survey period, 973 women were invited to participate in the survey, of which 700 women (72%) were able to be contacted. Of these women, 698 were eligible to participate and 514 (74% of women eligible and able to be contacted) completed the survey. The demographic characteristics of the sample are shown in Table 1.

**Table 1:** Characteristics of women and the maternity services they accessed (N = 514)

Characteristic	Mean (SD) or n (%)
Age	30 years (5 years)
Aboriginal, or Torres Strait Islander, or both	25 (5%)
Highest education level completed	
Completed high school or less	116 (23%)
Completed technical certificate or diploma	177 (34%)
Completed university or college degree or higher	221 (43%)
Index of disadvantage	
Most disadvantaged	220 (43%)
Mid disadvantaged	148 (29%)
Least disadvantage	146 (28%)
First pregnancy	202 (39%)
Singleton pregnancy	506 (98%)
Weeks pregnant at first public health service antental visit	19 week (6 weeks)
Pre-pregnancy BMI^a^	
< 18.5 kg/m^2^	19 (4%)
18.5–24.9 kg/m^2^	229 (49%)
25.0–29.9 kg/m^2^	119 (25%)
≥ 30.0 kg/m^2^	102 (22%)
Gestational diabetes mellitus during this pregnancy	62 (12%)
Pregnancy risk level	
Low risk	340 (66%)
Provider/s seen in antenatal visit	
Midwife only	354 (69%)
Doctor only	52 (10%)
Midwife and doctor	104 (20%)
Other provider involved	2 (0.4%)
Maternity service geographic remoteness	
Major city	420 (82%)

^a^ N = 469

### Reported receipt of guideline recommended antenatal care for GWG

Reported receipt of the individual care elements (assess, advise and refer) are presented in Table 2. One quarter of participants reported having been assessed at their first antenatal visit, including the practice elements of pre-pregnancy weight assessed (self-report or taken from medical record or referral documentation; n = 267/451, 59%), current weight measured (or offered to be measured; n = 280/451, 62%) and height measured (or offered to be measured; n = 196/451, 44%). More than 40% of women reported that they were weighed at one or more subsequent antenatal visit. Only 13% of women reported GWG assessment at both the first and one/more subsequent visits. Women were more likely to report assessment at a subsequent antenatal visit than at their first antenatal visit (*p* < 0.001). Around one third of women reported to have received advice for GWG, dietary intake and physical activity at their first visit. Less than 7% of women reported receipt of all assess and advice elements of guideline recommended antenatal care for GWG. One in ten women reported being referred to other services for additional GWG support at any visit. The most common referrals were to a dietitian (n = 29), a telephone coaching service (Get Healthy in Pregnancy (GHiP) [29]) (n = 13), and a diabetes educator (n = 3).

**Table 2:** Women’s reported receipt of assessment and care for GWG at initial and subsequent antenatal visits (N = 514)

Element of GWG care received	n	%	95% CI
**Assessment at first and subsequent antenatal visits** ^a^	59	13.1	10.3–16.5
Assessment at first antenatal visit ^a, b^	115	25.5	21.5–29.5
Assessment at subsequent antenatal visit	213	41.4	37.2–45.7
**All advice elements at first antenatal visit**	**154**	**30.0**	**26.0–33.9**
Advice on recommended GWG range	240	46.7	42.4–51.0
Advice on physical activity	248	48.3	43.9–52.6
Advice on dietary intake	322	62.7	58.5–66.8
**All assessment and advice for GWG** ^**c**^	**34**	**6.6**	**4.8–9.1**
**Referral to other specialist services at any visit**	**51**	**9.9**	**7.6–12.8**

^a^ Total N = 451

^b^ 148 participants responded ‘I do not remember’ to individual questions related to assessment at the first antenatal visit

^c^ Assessment at first and subsequent antenatal visits, and all advice elements at first antenatal visit

### Acceptablity of guideline recommended antenatal care for GWG

**Fig. 1:**
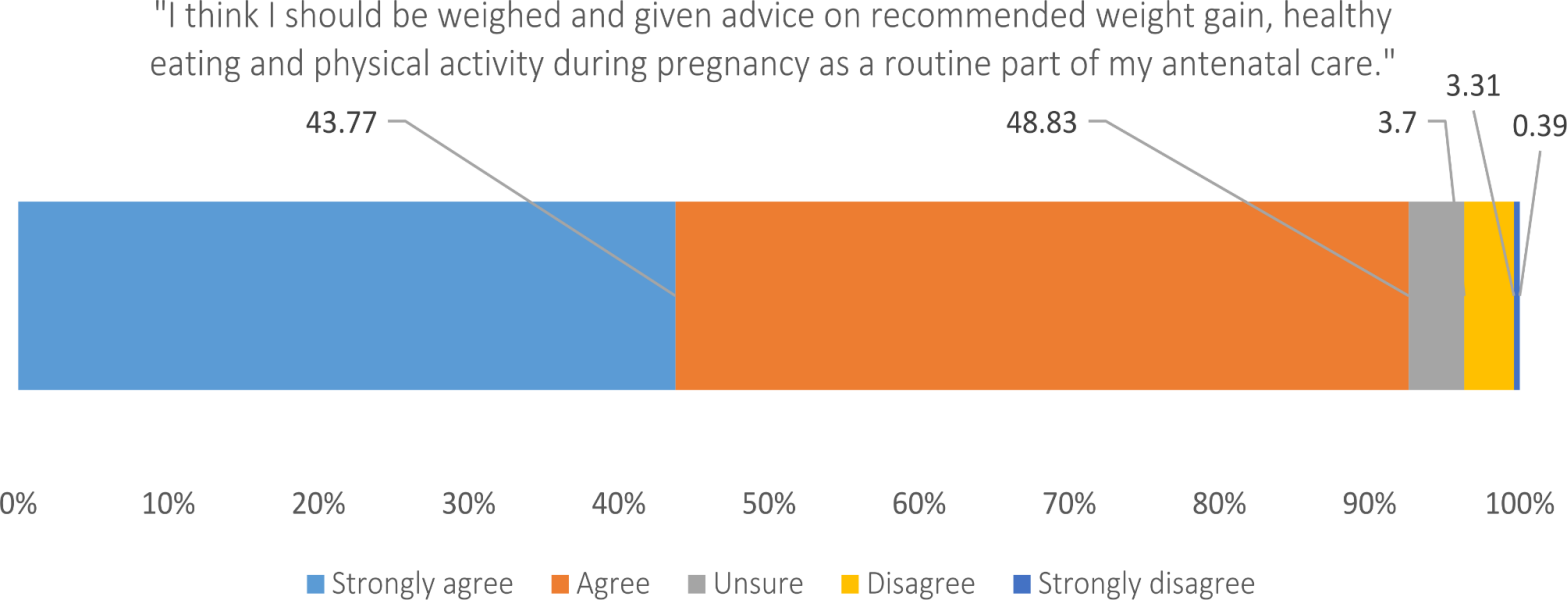
Women’s acceptability of recommended assessment and care for GWG

Women reported high acceptability for routine weighing and the provision of advice during routine antenatal care (Fig. 1). 92% of Aboriginal women and 93% of non-Aboriginal women strongly agreed or agreed that recommended care for GWG is acceptable.

Of the 7% (n = 38) of women that were unsure, disagreed or strongly disagreed that they should be weighed and given advice as part of routine care (Fig. 1), almost all strongly agreed or agreed with the statement that recommended care was acceptable when provided with conditional woman-centred care statements. These included if the antenatal care provider: weighed them and provided advice in a private room (n = 37) approached the conversation in a sensitive non-judgemental way (n = 35), explained that it would be important for their health and their baby (n = 34), asked for consent to discuss the topic first (n = 33) and if the woman knew they would be supported to gain weight within recommendations (n = 33).

### Associations between reported receipt and acceptability of guideline recommended care for GWG and charactersistics of pregnant women and maternity services

Associations between maternal and maternity service characteristics and receipt of recommended care elements are presented in Table 3. Women who identified as Aboriginal or Torres Strait Islander had increased odds of being assesssed at the first and subsequent antenatal visits (OR = 3.98, 95%CI: 1.09–14.52) and of receiving all assess and advice elements of care (OR = 24.54; 95%CI: 4.98-120.94). Women who lived in a least disadvantaged area had higher odds of receiving all assess and advice elements of care compared to women living in mid disadvantaged (OR = 18.5, 95%CI: 2.6-130.5) and most disadvantaged areas (OR = 13.1, 95%CI: 2.09–82.4). A woman experiencing her first pregancy had increased odds of receiving all advice at the first antenatal visit (OR = 2.34; 95%CI: 1.43–3.83) and receiving all assessment and advice (OR = 3.36; 95%CI: 1.27–8.86). Having a multiple pregnancy (e.g. twins) was associated with greater odds of receiving all advice components at the first antenatal visit (OR = 9.49; 95%CI: 1.71–52.68) and being referred to other support services (OR = 8.24; 95%CI: 1.45–46.93). A higher pre-pregnancy BMI increased the odds of being assessed at the first and subsequent antenatal visits (OR = 1.06; 95%CI:1.01–1.11), receiving all advice components at the first antenatal visit (OR = 1.04; 95%CI: 1.00-1.08), being referred to other support services at any visit (OR = 1.06; 95%CI: 1.01–1.11), and receiving all assessment and advice elements for GWG (OR = 1.13; 95%CI: 1.05–1.21). Being diagnosed with gestational diabetes mellitus during pregnancy was associated with higher odds of being referred to other support services (OR = 3.28; 95%CI: 1.40–7.68).

**Table 3:** Associations (n = 411) between reported receipt of assessment, all advice, referral to other support services, and all assess and advice elements of care and maternal and maternity service characteristics (n = 514)

Characteristics		Assessment at first and subsequent antenatal visits			All advice at first antenatal visit				Referral to other support services at any visit			All antenatal care (assessment at first and subsequent antenatal visits, and all advice)		
		n (%) or mean (SD)	Adjusted OR (95% CI)	*p*-value	n (%) or mean (SD)	Adjusted OR (95% CI)	*p*-value	n (%) or mean (SD)		Adjusted OR (95% CI)	*p*-value	n (%) or mean (SD)	Adjusted OR (95% CI)	*p*-value
Age, years		29.32 (5.31)^a^	0.96 [0.89–1.03]	0.26	29.50 (5.07)^b^	0.95 [0.90-1.00]	**0.046**	30.22 (5.16)^c^		0.95 [0.87–1.03]	0.18	28.62 (4.98)^d^	0.90 [0.81-1.00]	**0.041**
Aboriginal or Torres Strait Islander, or both														
	Yes	9 (40.91%)	3.98 [1.09–14.52]	**0.036**	11 (44.00%)	2.91 [0.94–8.99]	0.06	3 (12.00%)		0.53 [0.05–5.48]	0.59	7 (28.00%)	24.54 [4.98-120.94]	**< 0.001**
	No (referent)	50 (11.66%)			143 (29.24%)			48 (9.82%)				27 (5.52%)		
Education level				0.07			0.84				0.49			0.09
	Completed high school certificate or less	19 (18.63%)	2.77 [1.16–6.57]		37 (31.90%)	1.14 [0.60–2.16]		10 (8.62%)		1.06 [0.37–3.04]		12 (10.34%)	3.63 [1.10-11.95]	
	Completed technical certificate or diploma	26 (16.99%)	1.81 [0.82–4.02]		59 (33.33%)	1.17 [0.68–2.01]		28 (15.82%)		1.62 [0.69–3.79]		14 (7.91%)	1.51 [049-4.72]	
	Completed university or college degree or higher (referent)	14 (7.14%)			58 (26.24%)			13 (5.88%)				8 (3.62%)		
Area index of advantage				0.050			0.52				0.32			**0.011**
	Most disadvantaged	35 (17.86%)	1.53 [0.64–3.64]		67 (30.45%)	0.78 [0.42–1.45]		20 (9.09%)		0.46 [0.16–1.31]		22 (10.00%)	0.71 [0.23–2.22]	
	Mid disadvantaged	10 (7.81%)	0.47 [0.17–1.32]		42 (28.38%)	0.70 [0.38–1.31]		16 (10.81%)		0.88 [0.33–2.32]		2 (1.35%)	0.05 [0.01–0.38]	
	Least disadvantaged (referent)	14 (11.02%)			45 (30.82%)			15 (10.27%)				10 (6.85%)		
First pregnancy														
	Yes	23 (13.14%)	1.06 [0.53–2.11]	0.87	82 (40.59%)	2.34 [1.43–3.83]	**< 0.001**	24 (11.88%)		1.41 [0.65–3.08]	0.38	19 (9.41%)	3.36 [1.27–8.86]	**0.014**
	No (referent)	36 (13.04%)			72 (23.08%)			27 (8.65%)				15 (4.81%)		
Singleton pregnancy														
	No	1 (14.29%)	0.75 [0.08–7.18]	0.81	5 (62.50%)	9.49 [1.71–52.68]	**0.010**	48 (9.49%)		8.24 [1.45–46.93]	**0.018**	33 (6.52%)	6.19 [0.48–79.84]	0.16
	Yes (referent)	58 (13.06%)			149 (29.45%)			3 (37.50%)				1 (12.50%)		
Weeks pregnant at first antenatal visit		17.59 (6.37)^j^	0.94 [0.89-1.00]	0.07	19.94 (5.81)^k^	1.07 [1.02–1.11]	**0.003**	18.18 (6.14)^l^		1.00 [0.93–1.07]	0.90	18.82 (6.96)^m^	1.05 [0.97–1.14]	0.20
Pre-pregnancy BMI, kg/m^2^		29.73 (7.72)^e^	1.06 [1.01–1.11]	**0.021**	27.13 (7.18)^f^	1.04 [1.00-1.08]	**0.027**	29.69 (9.28)^g^		1.06 [1.01–1.11	**0.022**	31.19 (8.99)^h^	1.13 [1.05–1.21]	**< 0.001**
Gestational diabetes mellitus during this pregnancy														
	Yes	11 (19.30%)	1.78 [0.72–4.37]	0.21	19 (30.65%)	0.78 [0.37–1.63]	0.51	17 (27.42%)		3.28 [1.40–7.68]	**0.006**	5 (8.06%)	0.59 [0.15–2.25]	0.44
	No / don’t know (referent)	48 (12.18%)			135 (29.87%)			34 (7.52%)				29 (6.42%)		
Pregnancy risk level														
	Low risk	37 (12.09%)	1.32 [0.56–3.12]	0.53	104 (29.80%)	1.21 [0.65–2.25]	0.55	27 (7.74%)		0.74 [0.30–1.80]	0.51	21 (6.02%)	2.44 [0.67–8.92]	0.18
	High risk (referent)	22 (15.17%)			50 (30.30%)			24 (14.55%)				13 (7.88%)		
Provider/s seen in antenatal visit				0.50			0.30				0.45			0.21
	Doctor only	9 (18.00%)	1.78 [0.58–5.52]		17 (32.69%)	1.46 [0.60–3.55]		10 (19.61%)		2.43 [0.76–7.76]		6 (11.54%)	5.44 [1.11–26.74]	
	Midwife and doctor	11 (12.22%)	0.72 [0.28–1.82]		31 (29.81%)	1.86 [0.98–3.53]		15 (14.42%)		1.84 [0.72–4.71]		6 (5.77%)	1.29 [0.36–4.61]	
	Midwife only (referent)	39 (12.66%)			105 (29.66%)			26 (7.34%)				22 (6.21%)		
	Other provider involved	0 (0.00%)	N/A^i^		1 (50.00%)	0.92 [0.04–24.02]		0 (0%)		N/A^i^		0 (0.00%)	N/A^i^	
Maternity service location														
	Regional or rural	19 (22.09%)	1.03 [0.45–2.37]	0.95	31 (32.98%)	1.23 [0.62–2.44]	0.55	12 (12.77%)		1.92 [0.66–5.60]	0.23	14 (14.89%)	2.14 [0.70–6.61]	0.18
	Major city (referent)	40 (10.96%)			123 (29.29%)			39 (9.29%)				20 (4.76%)		

^a^ Note that only those who were assessed (N = 59) are presented here. Not assessed: Age: N = 392, M = 30.44 (4.72)

^b^ Note that only those who received all advice (N = 154) are presented here. Have not received all advice: Age: N = 360, M = 30.82 (4.83)

^c^ Note that only those who were referred (N = 51) are presented here. Not referred: Age: N = 463, M = 30.44(4.92)

^d^ Note that only those who received all assess and advice elements of care (N = 34) are presented here. Did not receive all assess and advice elements of care: Age: N = 480 M = 30.55(4.91)

^e^ Note that only those who were assessed (N = 50) are presented here. Not assessed: BMI: N = 362, M = 26.00 (5.97)

^f^ Note that only those who received all advice (N = 135) are presented here. Have not received all advice: BMI: N = 334, M = 25.85 (5.88)

^g^ Note that only those who were referred (N = 47) are presented here. Not referred: BMI: N = 422, M = 29.69 (9.28)

^h^ Note that only those who received all assess and advice elements of care (N = 29) are presented here. Did not receive all assess and advice elements of care: BMI: N = 440, M = 25.89(5.95)

^i^ Not computed as sample size too small

^j^ Note that only those who were assessed (N = 59) are presented here. Not assessed: Weeks pregnant at first visit: N = 392, M = 19.29 (5.68)

^k^ Note that only those who received all advice (N = 132) are presented here. Have not received all advice: Weeks pregnant at first visit: N = 319, M = 18.71 (5.81)

^l^ Note that only those who were referred (N = 44) are presented here. Not referred: Weeks pregnant at first visit: N = 407, M = 19.17 (5.76)

^m^ Note that only those who received all assess and advice elements of care (N = 34) are presented here. Did not receive all assess and advice elements of care: Weeks pregnant at first visit: N = 417, M = 19.09(5.70)

Being pregnant for the first time increased the odds of women reporting recommended GWG care as part of routine antenatal care to be acceptable (OR = 2.98; 95% CI:1.13–7.86) (Table 4). No other maternal or service characteristics were associated with acceptability of recommended GWG care.

**Table 4:** Multivariate associations between women’s acceptability of recommended GWG care and maternal and maternity service characteristics (n = 514)

Characteristics		Acceptability of recommended GWG care (agree/strongly agree) as part of routine antenatal care			
		N (%) or mean (SD)	Adjusted OR (95% CI) N = 411	*p*-value	
Age, years		30.34 (5.02) (n = 476)	0.98 [0.90–1.07]	0.71	
Aboriginal or Torres Strait Islander, or both					
	Yes	23 (92.00%)	0.66 [0.13–3.38]	0.62	
	No (referent)	453 (92.64%)			
Education level				0.42	
	Completed high school certificate or less	110 (94.83%)	2.21 [0.67–7.29]		
	Completed technical certificate or diploma	165 (93.22%)	1.34 [0.55–3.24]		
	Completed university or college degree or higher (referent)	201 (90.95%)			
Area index of advantage				0.73	
	Most disadvantaged	206 (93.64%)	1.09 [0.37–3.19]		
	Mid disadvantaged	136 (91.89%)	0.75 [0.28–2.02]		
	Least disadvantaged (referent)	134 (91.78%)			
First pregnancy					
	Yes	193 (95.54%)	2.98 [1.13–7.86]	**0.027**	
	No (referent)	283 (90.71%)			
Singleton pregnancy					
	Yes (referent)	469 (92.69%)			
	No	7 (87.50%)	0.50 [0.05–4.80]	0.55	
Weeks pregnant at first antenatal visit		19.07 (5.88) (n = 416)	0.99 [0.92–1.06]	0.73	
Pre-pregnancy BMI, kg/m^2^		26.09 (6.26) (n = 435)	0.98 [0.92–1.05]	0.59	
Gestational diabetes mellitus during this pregnancy					
	Yes	58 (93.55%)	1.05 [0.32–3.45]	0.94	
	No / don’t know (referent)	418 (92.48%)			
Pregnancy risk level					
	Low risk	327 (93.70%)	1.98 [0.74–5.29]	0.17	
	High risk (referent)	149 (90.30%)			
Provider/s seen in antenatal visit				0.90	
	Doctor only	48 (92.31%)	1.54 [0.37–6.32]		
	Midwife and doctor	97 (93.27%)	1.46 [0.49–4.31]		
	Midwife only (referent)	328 (92.66%)			
	Other provider involved	2 (100%)	N/A	0.99	
Maternity service location				0.74	
	Regional or rural	89 (94.68%)	1.24 [0.34–4.49]		
	Major city (referent)	387 (92.14%)			

### Prevalence of GWG within/outside of recommended ranges and recall of antenatal care provider recommendations

**Fig. 2:**
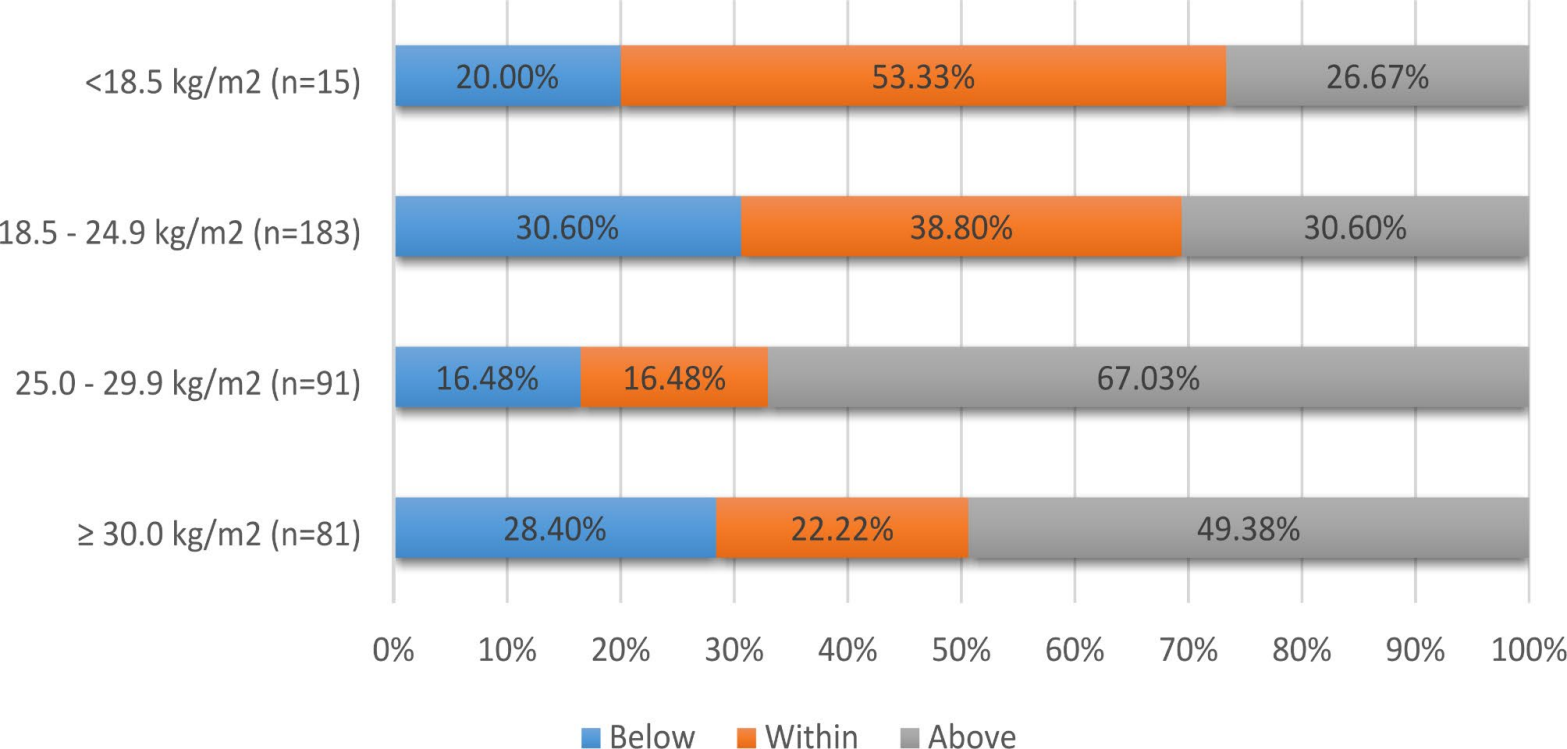
Proportion of women with singleton pregnancies who reported total GWG at birth below, within and above recommendations (N = 370)

Of the 370 women with singleton pregnancies who could recall their pre-pregnancy weight, height and weight prior to birth, 258 (70%) reported GWG below (26%) or above (44%) the guideline recommendations. The percentages of women who gained weight below, within and above the GWG recommendations for each pre-pregnancy BMI category are presented in Fig. 2.

**Fig. 3:**
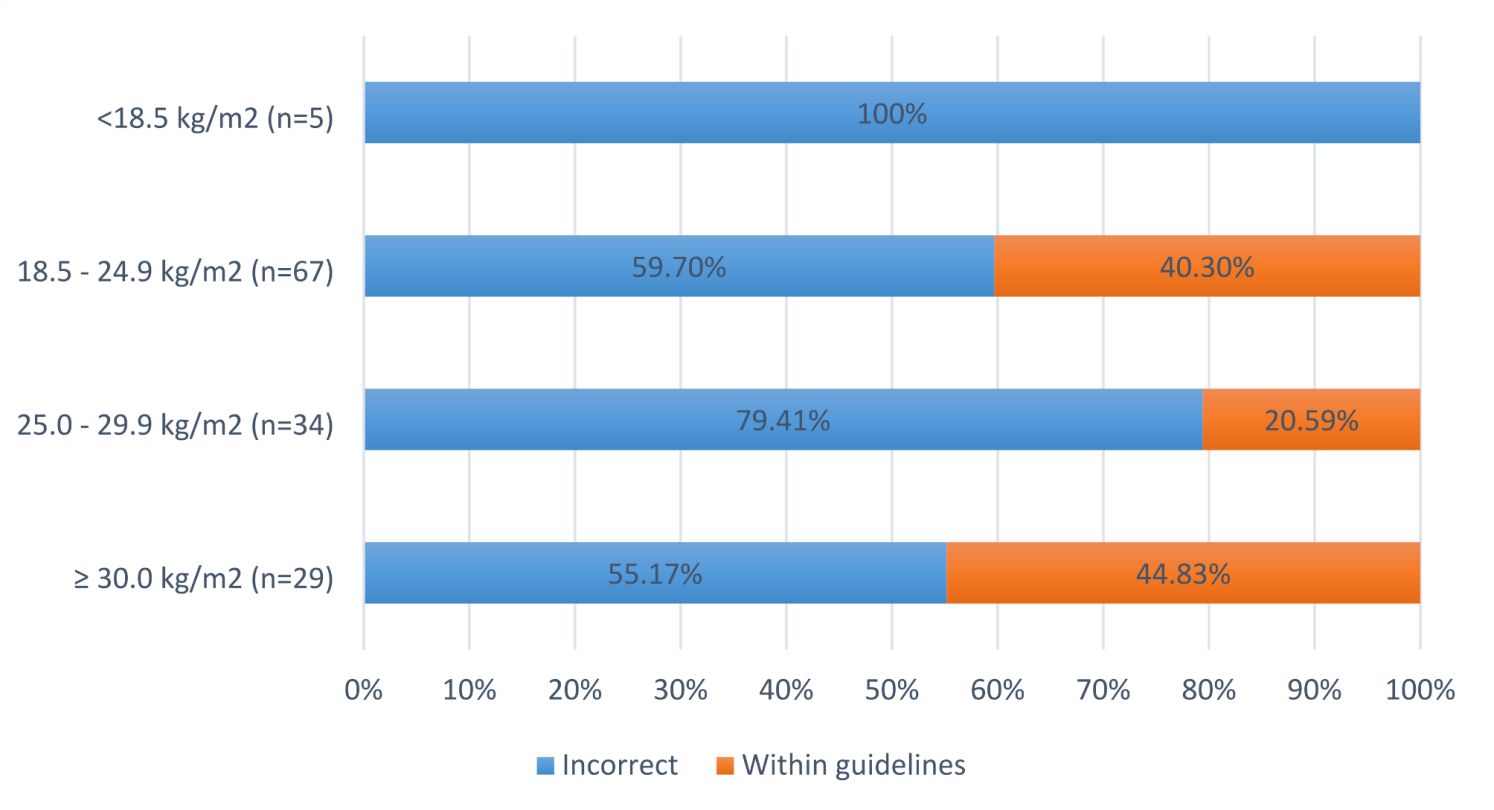
Proportion of women with singleton pregnancies (who could recall GWG recommendations) who reported a GWG recommendation that was incorrect or within recommended ranges based on their pre-pregnancy BMI (N = 135)

One hundred and thirty-five women (36%) could recall the weight they were recommended to gain by their antenatal care provider. For 88 (24%) of these women, the weight or weight range that they reported was within guideline recommendations based on their pre-pregnancy BMI. Women with a pre-pregancy BMI ≥ 30.0 kg/m^2^ (n = 29) had the highest proportion of women who reported a GWG recommendation that was correct based on their pre-pregnancy BMI (45%) (Fig. 3).

### Preference for GWG support

Almost all women (97%) reported that gaining recommended weight during pregnancy was important to them. Most women reported wanting to receive support for GWG: during existing antentatal appointments (94%), through brochures they can refer to later (85%), through referrals to talk with other health professionals in a face-to-face visit (76%) or telephone-based service (66%), or other modes of support (25%). Using the open field response to indicate preference for ‘other support methods’, women commonly requested emails (n = 43), websites (n = 37), mobile phone applications (n = 18), and group education classes ( n = 8) as another preferred mode of receiving further information and support.

## Discussion

This is the first comprehensive study of pregnant women’s reported receipt and acceptability of guideline recommended care for GWG, and factors associated with receiving such care, in the Australian public antenatal care setting [8, 10, 20, 23, 30, 31]. Only 7% of women reported receipt of both assessment and advice components at their antenatal visits. Less than 10% of participants were referred to additional support services (such as dietitians), despite 70% of women reporting to gain weight outside of recommended weight gain ranges by the end of their pregnancy. Most women agreed that the provision of recommended care for GWG is acceptable. Women who were younger, identifying as Aboriginal or Torres Strait Islander, had a higher pre-pregnancy BMI, in a first pregnancy, and living in an area of least disadvantage had higher odds of receiving the assess and advise components of recommended care. Recommended antenatal care for GWG is not universally provided and practice-change strategies in the antenatal setting are required to ensure all pregnant women routinely receive recommended GWG care.

Just 13% of women reported receiving all assess elements of care at their first and at a subsequent antenatal visit. These findings are comparable with those from an Australian prospective observational study of 492 women which reported that 64.6% of women were rarely or never weighed [23], and an retrospective study examining weight record data in an electronic medical record system which showed than only 4.2% of women had a weight recorded at each antenatal visit [15]. In February 2018, six months prior to the commencement of this study, the Australian Clinical Practice Guidelines changed the practice recommendations for GWG assessment from routine weighing women where clinically indicated [32] to routine weighing of all women at all appointments [6]. A web-based approach was used to disseminate the revised Clinical Practice Guidelines [6], and such a passive distribution method may have resulted in slow and low uptake [33–35] of routine weighing by antenatal care providers.

Less than one in three women reported receiving all elements of recommended advice for GWG during their first antenatal visit, with more women reporting dietary intake advice (63%), than physical activity advice (48%) and guidance on their recommended range of weight gain (47%). A systematic review [10] found higher levels of care provision for guidance on women’s recommended range of weight gain (69%), but did not report care for dietary intake or physical activity advice, or receipt of all advice elements with which to compare our study findings to. Of further concern, accuracy of the recommended weight gain range reported by women is suboptimal. Only 65% of women in our study (who could recall the weight they were recommended to gain) and 50% of women from a previous systematic review [10] reported receiving weight gain recommendations consistent with the IOM guidelines [4]. Recall bias is a possible explanation for the high levels of inaccurate GWG ranges recalled by pregnant women [7, 10], however, review evidence also suggests that a high proportion of health care providers are not able to recall correct GWG ranges, or use an inaccurate pre-pregnancy BMI to provide advice [7, 10].

The small proportion of women (10%) who were referred to other services may be reflective of the low levels of weight assessment at the first and ongoing antenatal visits. Without routine weight monitoring, women who are gaining weight at a rate below or above their recommendations are not able to be identified as needing referral to specialist services. Our referral findings are comparable with a US observational study that found that 10.6% of antenatal visits included any arrange/referral care [36]. Lower referrals may also reflect a lower prioritisation of GWG as a referring health issue, and a lack of public dietetic service capacity to provide timely referrals to address maternal health as found in other Australian research [37]. It has been suggested that care aspects like referring to other care services require more time and follow-through [36], better care provider education and guidance, and different organisational structures and more service capacity [37, 38]. Further research should investigate both pregnant women’s acceptability of, and antenatal care providers’ barriers to, offering GWG referrals to maximise acceptance and engagement. Integration of referral services into antenatal care and an increase in referral services capacity may improve the provision of GWG referrals [39].

A number of maternal and maternity service characteristics were found to increase the odds of receipt of recommended care for GWG, indicating that care is not provided routinely to all women by all antenatal care providers. Women who were younger and identify as Aboriginal or Torres Strait Islander were more likely to report receipt of all guideline care elements. These characteristics are similar to those reported by other antenatal care research for GWG and other preventive care topics [24, 40–42]. Women in their first pregnancy may be more likely to receive recommended care due to a health care provider’s belief that women who have previously received antenatal care for an earlier pregnancy would know how much weight to gain, and recommended dietary intake and physical activity behaviours [43]. A higher pre-pregnancy BMI has also previously been found to be associated with higher likelihood of being weighed at every antenatal visit [15, 17]. Antenatal care providers commonly express a misconception that GWG care is only important for women with obesity [17] despite evidence that all women are at risk of gaining weight outside of recommendations [1]. Having a higher pre-pregnancy BMI, multiple pregnancy, and gestational diabetes diagnosis were associated with greater odds of being referred to specialist services, such as a dietitian. These conditions often pose a higher pregnancy risk [44, 45] and referrals may relate to dietary management for gestational diabetes or ensuring adequate dietary intake for the growth of multiple foetuses, as well as for GWG.

Most Aboriginal and non-Aboriginal women report recommended care to be acceptable, with higher acceptability among women experiencing their first pregnancy. No other research was identified that explored Aboriginal Australian pregnant women’s acceptability of recommended GWG care, and more supporting evidence is needed using appropriate research methods [46–48]. Among the 7% of women who reported lower acceptability, their acceptability increased when person-centred care conditions were stipulated, including asking consent, ensuring privacy, sensitive non-judgemental communication, discussing its health importance, and providing ongoing support. Such high acceptability is consistent with qualitative [16, 49–51] and quantitative research finding that 80% of pregnant women consent to be weighed [49] and want to receive weight gain, diet and physical activity support [21, 52]. These findings on women-reported acceptability of recommended care are in contrast with research identifying barriers to care provision reported by antenatal care providers [7, 53, 54] who report a perception that pregnant women do not want to be weighed, and weight management conversations cause anxiety in women and negatively impact their rapport [7, 16, 17, 53]. Antenatal care providers’ concerns may be disproportionately influenced from their experiences of providing care to a small proportion of women reporting low acceptability, and other external factors such as broader weight stigma [55]. As most women reported a preference to receive care in existing appointments, initiatives to upskill antenatal care providers in person-centred approaches may improve their confidence and competence in providing, and pregnant women’s acceptability of, GWG care.

The findings of this study should be interpreted considering a number of strengths and limitations. The study includes a large sample of women from diverse socioeconomic backgrounds recruited from the public antenatal care setting. While only 52% of women invited to participate in the study completed the survey, the sample demographic characteristics are comparable to a large Australian data collection report [56]. Only 25 women who identified as Aboriginal or Torres Strait Islander participated in the study, therefore the findings should be interpreted with such consideration. The use of women’s self-report data limits any response bias associated with health providers’ self-report of care provision. However, women’s self-reported care receipt is influenced by recall bias based on participants’ ability to correctly remember past events, with this survey completed 1–5 months post pregnancy. This resulted in missing data and potential for incorrect recall, and the study findings should be interpreted with such consideration. Women’s self-reported care receipt, acceptability and anthropometrics measures may also be influenced by social desirability bias, with a tendency for people to over-report more socially acceptable attributes and behaviours [57–59]. It was not possible to determine if appropriate referrals were made, or assess referrals as part of recommended care, as no data was available indicating if women were gaining weight within recommendations at the time of each antenatal visits, which would indicate a need for referral based on the Clinical Practice Guidelines for Pregnancy Care [6, 32]. Future studies conducted throughout the pregnancy period should be conducted to reduce issues with participant recall and identify if recommended referrals are provided to women gaining weight outside of recommendations. The study did not collect information on previous live births or stillbirths and, as such, a woman’s first pregnancy is not able to be defined as either parity or gravidity. The study defined pregnancy risk according to the type and model of antenatal care that the woman received, which may not correspond with an individual’s actual medical risk. Future research could also examine associations between continuity of care models and receipt of recommended GWG care, which was unable to be investigated in this study. The study was limited to pregnant women aged 18 years and over who were proficient in English, had not experienced an adverse pregnancy outcome related to their recent pregnancy (miscarriage or stillbirth) and were receiving the majority of their antenatal care through a public maternity service within one health district in Australia. Therefore, the extent to which these findings are generalisable to other women and maternity services in Australia and internationally is unknown.

## Conclusions

Most women did not receive antenatal care for GWG as recommended by the Australian Clinical Practice Guidelines for Pregnancy Care, despite high acceptability of receiving such care. Care for GWG is not being delivered consistently to all women regardless of their personal characteristics or those of the service that they attend. There is a need for service-wide practice change to increase care for GWG, dietary intake and physical activity in pregnancy.

### Electronic supplementary material

Below is the link to the electronic supplementary material.

**Additional File 1:** Survey questions relating to antenatal care for gestational weight gain

## Data Availability

Data are available from the authors upon reasonable request and with permission from the Hunter New England Human Research Ethics Committee, Aboriginal Health and Medical Research Council, and the University of Newcastle Human Research Ethics Committee.

## List of abbreviations

BMI: Body Mass Index
GWG: Gestational weight gain
IOM: Institute of Medicine
OR: Odds Ratio

## References

[1] Goldstein RF, Abell SK, Ranasinha S, Misso M, Boyle JA, Black MH (2017). Association of gestational weight gain with maternal and infant outcomes: a systematic review and meta-analysis. JAMA.

[2] Phelan S (2010). Pregnancy: a teachable moment for weight control and obesity prevention. Am J Obstet Gynecol.

[3] Walker R, Bennett C, Blumfield M, Gwini S, Ma J, Wang F (2018). Attenuating pregnancy weight gain—what works and why: a systematic review and meta-analysis. Nutrients.

[4] Rasmussen KM, Yaktine AL, Institute of Medicine (US) National Research Council Committee to Reexamine IOM Pregnancy Weight Guidelines (2009). The National Academies Collection: reports funded by National Institutes of Health. Weight gain during pregnancy: reexamining the guidelines.

[5] Scott C, Andersen CT, Valdez N, Mardones F, Nohr EA, Poston L (2014). No global consensus: a cross-sectional survey of maternal weight policies. BMC Pregnancy Childbirth.

[6] Australian Government Department of Health (2018). Clinical practice guidelines: pregnancy care.

[7] Weeks A, Liu RH, Ferraro ZM, Deonandan R, Adamo KB (2018). Inconsistent weight communication among prenatal healthcare providers and patients: a narrative review. Obstet Gynecol Surv.

[8] Willcox JC, Campbell KJ, McCarthy EA, Lappas M, Ball K, Crawford D (2015). Gestational weight gain information: seeking and sources among pregnant women. BMC Pregnancy Childbirth.

[9] Olagbuji BN, Olofinbiyi BA, Akintayo AA, Aduloju OP, Ade-Ojo PI (2015). Maternal perspectives on gestational weight gain: critical information on developing weight control interventions. Nigerian Med Journal: J Nigeria Med Association.

[10] Whitaker KM, Becker C, Healy H, Wilcox S, Liu J (2021). Women’s report of health care provider advice and gestational weight gain: a systematic review. J Womens Health.

[11] Kominiarek MA, Gay F, Peacock N (2015). Obesity in pregnancy: a qualitative approach to inform an intervention for patients and providers. Matern Child Health J.

[12] Stengel MR, Kraschnewski JL, Hwang SW, Kjerulff KH, Chuang CH (2012). What my doctor didn’t tell me: examining health care provider advice to overweight and obese pregnant women on gestational weight gain and physical activity. Womens Health Issues.

[13] Swift JA, Pearce J, Jethwa P, Taylor MA, Avery A, Ellis S (2016). Antenatal weight management: women’s experiences, behaviours, and expectations of weighing in early pregnancy. J Pregnancy.

[14] Waring ME, Moore Simas TA, Barnes KC, Terk D, Baran I, Pagoto SL (2014). Patient report of Guideline-Congruent Gestational Weight Gain advice from prenatal care providers: differences by Prepregnancy BMI. Birth.

[15] Wilkinson S, Beckmann M, Donaldson E, McCray S (2019). Implementation of gestational weight gain guidelines-what’s more effective for ensuring weight recording in pregnancy?. BMC Pregnancy Childbirth.

[16] Olander EK, Atkinson L, Edmunds JK, French DP (2011). The views of pre- and post-natal women and health professionals regarding gestational weight gain: an exploratory study. Sex Reprod Healthc.

[17] Willcox JC, Campbell KJ, van der Pligt P. Excess gestational weight gain: an exploration of midwives’ views and practice. BMC Pregnancy Childbirth. 2012;12(102). 10.1186/1471-2393-12-102 PMC3531303 23013446

[18] Nikolopoulos H, Mayan M, MacIsaac J, Miller T, Bell RC (2017). Women’s perceptions of discussions about gestational weight gain with health care providers during pregnancy and postpartum: a qualitative study. BMC Pregnancy Childbirth.

[19] Baron R, Heesterbeek Q, Manniën J, Hutton EK, Brug J, Westerman MJ (2017). Exploring health education with midwives, as perceived by pregnant women in primary care: a qualitative study in the Netherlands. Midwifery.

[20] Knight-Agarwal CR, Williams LT, Davis D, Davey R, Shepherd R, Downing A (2016). The perspectives of obese women receiving antenatal care: a qualitative study of women’s experiences. Women Birth.

[21] de Jersey SJ, Nicholson JM, Callaway LK, Daniels LA (2013). An observational study of nutrition and physical activity behaviours, knowledge, and advice in pregnancy. BMC Pregnancy Childbirth.

[22] Kingsland M, Doherty E, Anderson AE, Crooks K, Tully B, Tremain D (2018). A practice change intervention to improve antenatal care addressing alcohol consumption by women during pregnancy: research protocol for a randomised stepped-wedge cluster trial. Implement Sci.

[23] de Jersey SJ, Nicholson JM, Callaway LK, Daniels LA (2012). A prospective study of pregnancy weight gain in a ustralian women. Aust N Z J Obstet Gynaecol.

[24] Doherty E, Wiggers J, Wolfenden L, Anderson AE, Crooks K, Tsang TW (2019). Antenatal care for alcohol consumption during pregnancy: pregnant women’s reported receipt of care and associated characteristics. BMC Pregnancy Childbirth.

[25] Australian Bureau of Statistics. SEIFA (2008). Socio-economic indexes for areas.

[26] SAS system for Windows. Copyright © 2011 SAS Institute Inc. SAS and all other SAS Institute Inc. product or service names are registered trademarks or trademarks of SAS Institute Inc., Cary, NC, USA.

[27] Department of Health and Aged Care (2001). Measuring remoteness: accessibility/remoteness index of Australia (ARIA).

[28] World Health Organization. Physical status: The use and interpretation of anthropometry. 1995.

[29] NSW Government. Get healthy [Available from: https://www.gethealthynsw.com.au/program/get-healthy-in-pregnancy/

[30] de Jersey S, Guthrie T, Callaway L, Tyler J, New K, Nicholson J (2022). A theory driven, pragmatic trial implementing changes to routine antenatal care that supports recommended pregnancy weight gain. BMC Pregnancy Childbirth.

[31] de Jersey S, Guthrie T, Tyler J, Ling WY, Powlesland H, Byrne C (2019). A mixed method study evaluating the integration of pregnancy weight gain charts into antenatal care. Matern Child Nutr.

[32] Australian Health Ministers’ Advisory Council (2012). Clinical practice guidelines: Antenatal care–module 1.

[33] Bero LA, Grilli R, Grimshaw JM, Harvey E, Oxman AD, Thomson MA (1998). Closing the gap between research and practice: an overview of systematic reviews of interventions to promote the implementation of research findings. BMJ.

[34] Lavis JN, Robertson D, Woodside JM, McLeod CB, Abelson J (2003). How can research organizations more effectively transfer research knowledge to decision makers?. Milbank Q.

[35] Prior M, Guerin M, Grimmer-Somers K (2008). The effectiveness of clinical guideline implementation strategies–a synthesis of systematic review findings. J Eval Clin Pract.

[36] Washington Cole KO, Gudzune KA, Bleich SN, Bennett WL, Cheskin LJ, Henderson JL (2017). Influence of the 5A’s counseling strategy on weight gain during pregnancy: an observational study. J Womens Health.

[37] Wilkinson SA, Donaldson E, Willcox J (2020). Nutrition and maternal health: a mapping of Australian dietetic services. BMC Health Serv Res.

[38] Fealy S, Hollis J, Martin J, Leigh L, Oldmeadow C, Collins CE (2022). Modeling the predictive value of evidence-based referral criteria to support healthy gestational weight gain among an Australian pregnancy cohort. Nutrients.

[39] Wilkinson SA, Donaldson E, McCray SJ (2018). Re-evaluating the nutritional awareness, knowledge and eating behaviours of women attending a tertiary maternity hospital following iterative service redesign. Nutr Diet.

[40] Cheng D, Kettinger L, Uduhiri K, Hurt L (2011). Alcohol consumption during pregnancy: prevalence and provider assessment. Obstet Gynecol.

[41] Stotland N, Tsoh JY, Gerbert B (2012). Prenatal weight gain: who is counseled?. J Womens Health.

[42] Yelland JS, Sutherland GA, Brown SJ (2012). Women’s experience of discrimination in Australian perinatal care: the double disadvantage of social adversity and unequal care. Birth.

[43] Lee A, Newton M, Radcliffe J, Belski R (2018). Pregnancy nutrition knowledge and experiences of pregnant women and antenatal care clinicians: a mixed methods approach. Women Birth.

[44] Buchanan TA, Xiang AH, Page KA (2012). Gestational Diabetes Mellitus: risks and management during and after pregnancy. Nat Reviews Endocrinol.

[45] Dodd JM, Crowther CA (2005). Evidence-based care of women with a multiple pregnancy. Best Pract Res Clin Obstet Gynecol.

[46] Harfield S, Pearson O, Morey K, Kite E, Canuto K, Glover K (2020). Assessing the quality of health research from an indigenous perspective: the Aboriginal and Torres Strait Islander quality appraisal tool. BMC Med Res Methodol.

[47] Huria T, Palmer SC, Pitama S, Beckert L, Lacey C, Ewen S (2019). Consolidated criteria for strengthening reporting of health research involving indigenous peoples: the CONSIDER statement. BMC Med Res Methodol.

[48] Jamieson LM, Paradies YC, Eades S, Chong A, Maple-Brown LJ, Morris PS (2012). Ten principles relevant to health research among indigenous Australian populations. Med J Aust.

[49] Allen-Walker V, Hunter A, Holmes V, McKinley M (2020). Weighing as part of your care: a feasibility study exploring the re-introduction of weight measurements during pregnancy as part of routine antenatal care. BMC Pregnancy Childbirth.

[50] Furness PJ, McSeveny K, Arden MA, Garland C, Dearden AM, Soltani H (2011). Maternal obesity support services: a qualitative study of the perspectives of women and midwives. BMC Pregnancy Childbirth.

[51] Daley A, Jolly K, Jebb S, Lewis A, Clifford S, Roalfe A (2015). Feasibility and acceptability of regular weighing, setting weight gain limits and providing feedback by community midwives to prevent excess weight gain during pregnancy: randomised controlled trial and qualitative study. BMC Obes.

[52] Vanstone M, Kandasamy S, Giacomini M, DeJean D, McDonald SD (2017). Pregnant women’s perceptions of gestational weight gain: a systematic review and meta-synthesis of qualitative research. Matern Child Nutr.

[53] Heslehurst N, Newham J, Maniatopoulos G, Fleetwood C, Robalino S, Rankin J (2014). Implementation of pregnancy weight management and obesity guidelines: a meta-synthesis of healthcare professionals’ barriers and facilitators using the theoretical domains Framework. Obes Rev.

[54] Stotland NEGP, Bogetz A, Harper CC, Abrams B, Gerbert B (2010). Preventing excessive weight gain in pregnancy: how do prenatal care providers approach counseling?. J Womens Health.

[55] Hill B, Rodriguez ACI, editors. Weight stigma across the preconception, pregnancy, and postpartum periods: a narrative review and conceptual model. Semin Reprod Med; 2020: Thieme Medical Publishers, Inc. 10.1055/s-0041-1723775 33728621

[56] Australian Institute of Health and Welfare. Australia’s mothers and babies. 2021.

[57] Burke MA, Carman KG (2017). You can be too thin (but not too tall): Social desirability bias in self-reports of weight and height. Econ Hum Biol.

[58] Hinkle SN, Sharma AJ, Schieve LA, Ramakrishnan U, Swan DW, Stein AD (2013). Reliability of gestational weight gain reported postpartum: a comparison to the birth certificate. Matern Child Health J.

[59] Stuart GS, Grimes DA (2009). Social desirability bias in family planning studies: a neglected problem. Contraception.

