# Supplementary material for: Antenatal care addressing gestational weight gain (GWG): a cross sectional study of pregnant women’s reported receipt and acceptability of recommended GWG care and associated characteristics
Source: BMC Pregnancy Childbirth. 2024 Feb 6;24:111. doi: 10.1186/s12884-023-06158-4 (PMC10845753; doi:10.1186/s12884-023-06158-4)
Supplement: Supplementary file 1 — Additional File 1: Survey questions relating to antenatal care for gestational weight gain [file 12884_2023_6158_MOESM1_ESM.docx]

**Supplementary File 1. Survey questions relating to antenatal care for gestational weight gain**

The purpose of this short survey is to find out about your experiences during pregnancy, and the care you may have received during your antenatal visits, in relation to weight gain and smoking in pregnancy.

All of your answers are confidential and will not identify you. Completing the survey will have no impact on any care you receive from our services. Your feedback will be used to make sure our services are providing the best quality of care.

**Instructions**

Questions should be answered by selecting the relevant box. There are no right or wrong answers for this survey - it is your honest answers that are most important. The survey is voluntary and if your feel uncomfortable at any time you can stop the survey.

*Thank you for taking the time to complete this survey.*

| *The following questions ask about your antenatal experiences in relation to your most recent pregnancy.* | | | | | |
| --- | --- | --- | --- | --- | --- |
|  | | | | | |
| 1. **How many weeks’ pregnant were you at the time of your baby’s birth?** | | | | | |
| (Open question) __________________________________________________ | | | | | |
|  | | | | | |
| 1. **My most recent pregnancy was a:** | | | | | |
| Single pregnancy | | | |  | |
| Multiple pregnancy (e.g. twins, triplets) | | | |  | |
|  | | | | | |
| 1. **Did you have gestational diabetes during your last pregnancy?** | | | | | |
| Yes | | | |  | |
| No | | | |  | |
| I do not remember | | | |  | |
| I would prefer not to answer | | | |  | |
|  | | | | | |
| *The following questions ask about your height and your weight at different times during your most recent pregnancy.* | | | | | |
|  | | | | | |
| 1. **What is your height? (report in cm or inches)** | | | | | |
| (Open question) __________________________________________________ | | | | | |
| I do not remember | |  | I would prefer not to answer | |  |
|  | | | | | |
| 1. **How much did you weigh before you became pregnant? (report in kg or pounds)** | | | | | |
| (Open question) __________________________________________________ | | | | | |
| I do not remember | |  | I would prefer not to answer | |  |
|  | | | | | |
| 1. **How much did you weigh at your booking in visit *(your first comprehensive antenatal assessment)?* at the hospital? (report in kg or pounds)** | | | | | |
| (Open question) __________________________________________________ | | | | | |
| I do not remember |  | | I would prefer not to answer | |  |
|  | | | | | |
| 1. **How much did you weigh at the time of your baby’s birth?** | | | | | |
| (Open question) __________________________________________________ | | | | | |
| I do not remember | |  | I would prefer not to answer | |  |
|  | | | | | |
| *The following questions ask about the care you received during your antenatal visits at the hospital.* | | | | | |
|  | | | | | |
| 1. **How many weeks pregnant were you at your booking in visit *(your first comprehensive antenatal assessment)* at the hospital?** | | | | | |
| (Open question) __________________________________________________ | | | | | |
| I do not remember | |  | I would prefer not to answer | |  |
|  | | | | | |
| 1. **Which health professional/s did you see at your booking in visit *(your first comprehensive antenatal assessment)* at the hospital? This would have taken 1.5-2 hours and you were asked a lot of questions. (Select all that apply)** | | | | | |
| Midwife | | | |  | |
| Aboriginal and Torres Strait Islander health worker | | | |  | |
| Multicultural health worker | | | |  | |
| General practitioner (GP) | | | |  | |
| Hospital doctor (specialist, obstetrician, Registrar, Junior Medical Officer) | | | |  | |
| I do not remember | | | |  | |
| I would prefer not to answer | | | |  | |
|  | | | | | |
| **At your booking in visit *(your first comprehensive antenatal assessment)* at the hospital:** | | | | | |
| 1. **Was your height recorded?** | | | | | |
| Yes, my height was measured during the visit | | | |  | |
| Yes, I told them my height during the visit | | | |  | |
| Yes, because they already had it on file (e.g. GP referral letter, patient medical record) | | | |  | |
| No | | | |  | |
| I do not remember | | | |  | |
|  | | | | | |
| 1. **Did a health professional weigh you?** | | | | | |
| Yes | | | |  | |
| No | | | |  | |
| I do not remember | | | |  | |
|  | | | | | |
| *If answered ‘No’ to question 11, proceed to question 12. If answered ‘Yes’ or ‘I do not remember’ to question 11, proceed to question 14.* | | | | | |
|  | | | | | |
| 1. **Did a health professional offer to weigh you?** | | | | | |
| Yes, but I chose not to be weighed | | | |  | |
| Yes, but they did not weigh me | | | |  | |
| No, but they recorded my weight from my medical record (e.g. GP referral letter, patient file) | | | |  | |
| No | | | |  | |
| I do not remember | | | |  | |
|  | | | | | |
| *If answered ‘Yes, but I chose not to be weighed’ to question 12, proceed to question 13. If answered any other response to question 12, proceed to question 14.* | | | | | |
|  | | | | | |
| 1. **What was the reason you chose not to be weighed?** | | | | | |
| (Open question) __________________________________________________ | | | | | |
| I do not remember | |  | I would prefer not to answer | |  |
|  | | | | | |
| 1. **Were you asked how much you weighed before you became pregnant?** | | | | | |
| Yes | | | |  | |
| No | | | |  | |
| I do not remember | | | |  | |
|  | | | | | |
| 1. **Did the health professional discuss the amount of weight you were recommended to gain during your pregnancy?** | | | | | |
| Yes | | | |  | |
| No | | | |  | |
| I do not remember | | | |  | |
|  | | | | | |
| *If answered ‘Yes’ to question 15, proceed to question 16. If answered ‘No’ or ‘I do not remember’ to question 15, proceed to question 17.* | | | | | |
|  | | | | | |
| 1. **How much total weight did the health professional recommended you gain during pregnancy? (report in kg or pounds)** | | | | | |
| (Open question) __________________________________________________ | | | | | |
| I do not remember | |  | I would prefer not to answer | |  |
|  | | | | | |
| 1. **Did the health professional explain the reason for measuring weight and weight gain during pregnancy?** | | | | | |
| Yes | | | |  | |
| No | | | |  | |
| I do not remember | | | |  | |
| I would prefer not to answer | | | |  | |
|  | | | | | |
| 1. **Did the health professional give you information about physical activity to support a healthy weight gain during pregnancy?** | | | | | |
| Yes | | | |  | |
| No | | | |  | |
| I do not remember | | | |  | |
| I would prefer not to answer | | | |  | |
|  | | | | | |
| 1. **Did the health professional give you information about healthy eating to support a healthy weight gain during pregnancy?** | | | | | |
| Yes | | | |  | |
| No | | | |  | |
| I do not remember | | | |  | |
| I would prefer not to answer | | | |  | |
|  | | | | | |
| 1. **Were you weighed by a health professional at other follow-up antenatal visits during pregnancy (other than your booking in visit)?** | | | | | |
| Yes | | | |  | |
| No, although this was offered to me in follow-up visit/s | | | |  | |
| No, and his was not offered to me in follow-up visits | | | |  | |
| I do not remember | | | |  | |
| I would prefer not to answer | | | |  | |
|  | | | |  | |
| *To what extent do you agree or disagree with these statements. Please tick one option per question.* | | | | | |
|  | | | | | |
| 1. **Maintaining a healthy weight during pregnancy is important to me.** | | | | | |
| Strongly agree | | | |  | |
| Agree | | | |  | |
| Neither agree nor disagree | | | |  | |
| Disagree | | | |  | |
| Strongly disagree | | | |  | |
|  | | | | | |
| 1. **I think I should be weighed and given advice on recommended weight gain, healthy eating and physical activity during pregnancy as a routine part of my antenatal care.** | | | | | |
| Strongly agree | | | |  | |
| Agree | | | |  | |
| Neither agree nor disagree | | | |  | |
| Disagree | | | |  | |
| Strongly disagree | | | |  | |
|  | | | | | |
| **I think I should be weighed and given advice on recommended weight gain, healthy eating and physical activity during pregnancy if:** | | | | | |
| 1. **They felt that it was important for providing care for my health and the health of my baby.** | | | | | |
| Strongly agree | | | |  | |
| Agree | | | |  | |
| Neither agree nor disagree | | | |  | |
| Disagree | | | |  | |
| Strongly disagree | | | |  | |
|  | | | | | |
| 1. **They asked if I wanted to discuss the topic first.** | | | | | |
| Strongly agree | | | |  | |
| Agree | | | |  | |
| Neither agree nor disagree | | | |  | |
| Disagree | | | |  | |
| Strongly disagree | | | |  | |
|  | | | | | |
| 1. **They approached the topic in a sensitive, non-judgmental way.** | | | | | |
| Strongly agree | | | |  | |
| Agree | | | |  | |
| Neither agree nor disagree | | | |  | |
| Disagree | | | |  | |
| Strongly disagree | | | |  | |
|  | | | | | |
| 1. **They weighed me and provided advice in a private room.** | | | | | |
| Strongly agree | | | |  | |
| Agree | | | |  | |
| Neither agree nor disagree | | | |  | |
| Disagree | | | |  | |
| Strongly disagree | | | |  | |
|  | | | | | |
| 1. **If I knew that my health professional would support me to achieve a healthy weight gain throughout my pregnancy.** | | | | | |
| Strongly agree | | | |  | |
| Agree | | | |  | |
| Neither agree nor disagree | | | |  | |
| Disagree | | | |  | |
| Strongly disagree | | | |  | |
|  | | | | | |
| *The following questions asks about other services you have used, and that you would have preferred to receive support from, during you pregnancy to support you to gain a healthy weight.* | | | | | |
|  | | | | | |
| 1. **Did your health professional refer you to any other service/s to support you to gain a healthy weight in pregnancy? (select all that apply)** | | | | | |
| No | | | |  | |
| Get Healthy in Pregnancy (GHiP) Telephone Coaching Service | | | |  | |
| Aboriginal Medical Services (AMS e.g. Awabakal, TAMS, Biripi) | | | |  | |
| Dietitian | | | |  | |
| Exercise specialist (e.g. exercise physiologist) | | | |  | |
| Gym or other specialist pregnancy exercise program (e.g. pregnancy yoga) | | | |  | |
| Alternative therapies (e.g. Naturopath) | | | |  | |
| Other (please write) | | | | ____________________ | |
| Unsure of the name of the service | | | |  | |
| I do not remember | | | |  | |
|  | | | | | |
| 1. **How would you have preferred to receive information and advice about eating and physical activity to support a healthy weight gain during pregnancy? (select all that apply)** | | | | | |
| During existing antenatal visits | | | |  | |
| Through additional antenatal visits | | | |  | |
| During an existing parenting education class | | | |  | |
| Through an additional antenatal group class early in pregnancy | | | |  | |
| Referral to talk to other health professionals (e.g. dietitians, exercise physiologists) in a face-to-face visit | | | |  | |
| Referral to talk to other health professionals (e.g. dietitians, exercise physiologists) through telephone services | | | |  | |
| Brochures that I can refer to later | | | |  | |
| Online websites that I can refer to later | | | |  | |
| Other (please write) | | | | _____________________ | |
| I would prefer not to answer | | | |  | |
|  | | | | | |
| 1. **Please add any other comments you may have about the care you received regarding weight gain in your pregnancy.** | | | | | |
| (Open question) __________________________________________________ | | | | | |
|  | | | | | |
| *Thank you for taking the time to complete this survey.* | | | | | |
